# Using Sulfobutylated and Sulfomethylated Lignin as Dispersant for Kaolin Suspension

**DOI:** 10.3390/polym12092046

**Published:** 2020-09-08

**Authors:** Derya Yesim Hopa, Pedram Fatehi

**Affiliations:** 1 Department of Chemical Engineering, Lakehead University, Thunder Bay, ON P7B 5E1, Canada; dyhopa@lakeheadu.ca; 2 Department of Chemical Engineering, Afyon Kocatepe University, Afyonkarahisar 03200, Turkey; 3 State Key Laboratory of Biobased Material and Green Papermaking, Qilu University of Technology (Shandong Academy of Sciences), Jinan 250353, China

**Keywords:** lignin, biopolymer, sulfobutylation, sulfomethylation, QCM, zeta potential, dispersion

## Abstract

Kraft lignin is an abundant natural resource, but it is underutilized. In this study, sulfoalkylated lignin derivatives with similar charge densities but with different alkyl chain length were produced via sulfobutylation and sulfomethylation reactions. The contact angle studies revealed that sulfobutylated lignin (SBL) with longer alkyl chains had a higher hydrophobicity than sulfomethylated lignin (SML) did. The adsorption behavior of sulfoalkylated lignins was studied using a Quartz crystal microbalance with dissipation (QCM-D) on Al_2_O_3_ coated surface as representative of positively charged sites of kaolin particles. The results of adsorption studies showed that SBL deposited more greatly than SML did on the Al_2_O_3_ surface, and it generated a thicker but less viscoelastic adlayer on the surface. The adlayer thickness and configuration of molecules on the surface were also related to the zeta potential and stabilization performance of the polymers in the kaolin suspension system. The results also confirmed that both lignin derivatives were very effective in dispersing kaolin particles at neutral pH, and their effectiveness was hampered under alkaline or acidic pH.

## 1. Introduction

The properties of kaolin, such as particle size, chemical stability, non-abrasiveness, and its whiteness [1], make it a widely used industrial mineral with a variety of uses in paint, ceramic, and paper industries [2,3,4]. The stabilization of kaolin suspensions is of great importance in these manufacturing processes since a stable and homogeneous colloidal kaolin suspension affects the final properties of the products and economic aspects of the processes [5,6]. Kaolin particles that are platelet in shape with negative basal surface charges and positive edge charges have heterogeneous structures [7]. The heterogeneous structure of kaolin promotes its aggregation in concentrated aqueous suspensions [8].

Dispersants are used to prevent the aggregation challenge of kaolin particles by creating electrostatic/steric repulsive forces between particles [9]. Synthetic polymers containing long alkyl chains such as alkylammonium surfactants [10], and poly-electrodes, such as poly-acrylamides, poly-acrylic acrylates, polycarboxylates, and their derivatives are widely used as dispersants for stabilizing kaolin particles in suspensions [6,9,11,12,13,14]. However, as synthetic dispersants are oil-based, and they cause serious environmental and health-related issues arising from their poor biodegradability and their degradation intermediate products, there is a great demand for developing sustainable and biodegradable dispersants [15,16,17].

Lignin is a suitable candidate for the production of non-toxic, renewable, and sustainable dispersants as it is available in large quantities [17,18]. However, lignin is not suitable for direct use as a dispersant due to its limited solubility in water [19]. On the other hand, there are a variety of lignin modification methods available for improving the solubility of lignin, which have substantial impacts on the adsorption of lignin on a charged surface [12,20]. The grafting of lignin with sulfonate containing alkyl chains, such as sulfoalkylation, is an important route as it could enhance the surface activity, and the adsorption affinity of lignin on a charged surface, which may promote the dispersion performance of lignin [21,22]. In the past, sulfomethylation has been reported as an effective grafting method for inducing lignin a dispersant for coal-water slurry, cement, concrete, and dye systems due to its affinity in improving the hydrophilicity and charge density of lignin [19,23,24,25]. Despite its effectiveness, sulfomethylation requires the use of formaldehyde, which is a toxic chemical. In this respect, there is a great demand for developing an alternative sulfoalkylation reaction that can be more environmentally friendly. In this regard, sulfobutylation may be an appropriate method for converting kraft lignin into a dispersant for kaolin suspensions as this reaction proceeds in aqueous media [21,26]. Moreover, sulfobutylation is a simple one-step reaction that is a time and energy-saving process for lignin modification [21,26]. One objective of this study was to assess the sulfobutylation of kraft lignin to produce a dispersant for kaolin suspensions.

In this study, sulfobutylated and sulfomethylated kraft lignin derivatives were produced to have similar charge densities. Water contact angle measurements were conducted for lignin derivatives, as contact angle measurement is a widely used tool for understanding the interfacial properties of lignin and also for investigating the changes in lignin structure after chemical modifications [27,28,29]. Adsorption analysis was conducted for both lignin derivatives via the Quartz crystal microbalance with dissipation (QCM-D) analysis on the Al_2_O_3_ surfaces to understand the adsorption and deposition performance of lignin derivatives on the edge of kaolin particles as they have positive charges. As the behavior of kaolin particles is strongly affected by the pH of the dispersion [30], the impact of pH changes on the adsorption and dispersion performance of lignin derivatives were also studied in this work. In this research, sulfoalkylated lignin with similar charge densities but with two different sulfonate groups were produced, and their dispersion performance in kaolin suspensions was investigated for the first time. The main novelties of this work were (1) the generation of sulfobutylation of lignin, (2) the performance assessment of the product as a dispersant, and (3) the comparison of sulfobutylated and sulfomethylated lignin as dispersants for kaolin suspension.

## 2. Materials and Methods

### 2.1. Materials

Softwood kraft lignin (KL) was supplied by FPInnovations, Thunder Bay, ON, Canada. Kaolin powder was supplied by Old Hickory Clay Company, Hickory, KY, USA. Sodium metabisulfite (Na_2_S_2_O_5_) (99.0%), formaldehyde (CH_2_O) (37.0%), polydimethyldiallyl ammonium chloride (20.0 wt.%), sodium hydroxide (97.0%), hydrochloric acid (37.0%), sodium nitrate (99.0%), dimethyl sulphate (98.0%) sulfuric acid (98.0%), D_2_O isotopic purity (99.8%), trimethylsilylpropanoic acid (TSP) (99.8%), para-hydroxybenzoic acid (99.0%), silicon oil, poly (ethylene oxide) and 1,4–butane sultone (99.8%) were purchased from Sigma-Aldrich (Canada). Potassium chloride (99.0%) and potassium hydroxide solution (8 M) were purchased from Fisher Scientific (Canada). Cellulose acetate dialysis membrane (molecular weight cut-off of 1000 g/mol) was obtained from Spectrum Laboratories Inc., USA.

### 2.2. Surface Area Analysis

The surface area of kaolin particles was measured using a Quantachrome surface area analyzer, Nova2200e, under N_2_ atmosphere. Approximately, 0.05 g of kaolin was dried at 250 °C for 4 h. The total specific surface area of the samples was determined according to the Brunauer–Emmett–Teller (BET, Boynton Beach, FL, USA) method via Nitrogen adsorption and desorption isotherms [31].

### 2.3. Particle Size Analysis

In this test, 10 g of kaolin particles were added to 100 mL of deionized water and stirred at 300 rpm and 25 °C for 1 h. After stirring, the particle size analysis of the suspension was determined using a MasterSizer 3000 (Malvern Instruments, Worcestershire, UK), which was equipped with a light scattering detector. All the measurements were carried out at room temperature.

### 2.4. Sulfobutylation

**Figure 1:**
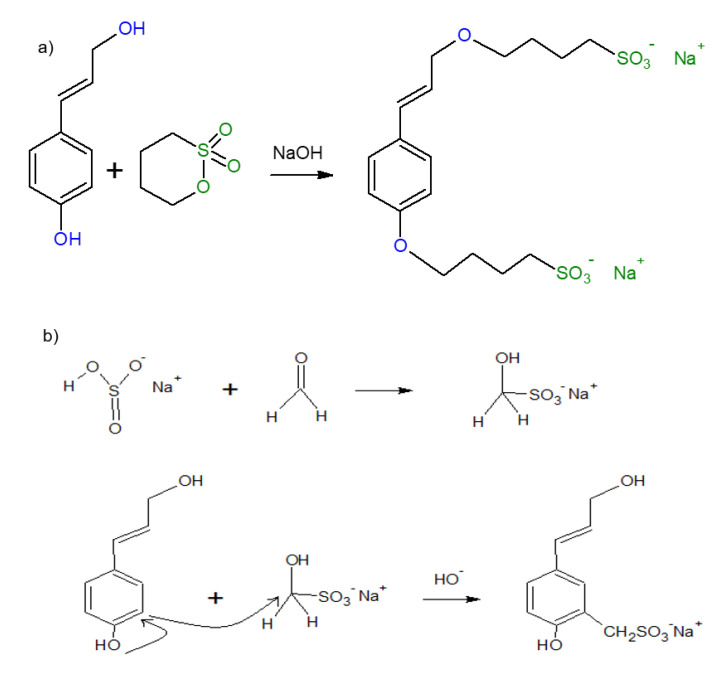
Reaction schemes of sulfonation of lignin (**a**) sulfobutylation of lignin via reacting phenolic and aliphatic –OH groups of kraft lignin with 1,4–butanesultone after ring-opening (**b**) sulfomethylation of lignin via formation of a sodium sulfonate methyl derivative.

The sulfobutylation of kraft lignin was carried out in a 100 mL three-neck round-bottom glass flask at 150 rpm and 70 °C for 3 h. A 1 g sample of softwood kraft lignin was dissolved in deionized water to prepare a 50 g/L lignin solution, while NaOH was used for adjusting the pH of the solution to 11.5–12. When lignin was completely dissolved, different ratios (0.02–1 mol/mol) of 1,4–butane sultone to lignin were prepared in the flask for reacting with phenolic and aliphatic –OH groups of kraft lignin. The reaction scheme of sulfobutylation of lignin is shown in Figure 1. Upon the reaction completion, the solution was cooled to room temperature, and its pH was adjusted to 7 using 1M sulfuric acid. Unreacted 1,4–butane sultone and ions were separated from sulfobutylated kraft lignin via dialysis, while water was changed every 12 h for 2 days. The dialyzed sulfobutylated kraft lignin was then dried at 105 °C in an oven overnight and stored at 4 °C for further use. Based on the outcomes of the charge density analysis, the sulfobutylated lignin that was generated using 0.2 mol/mol 1,4–butane sultone/lignin ratio for 3 h at 70 °C was selected as the best sample (SBL) for further characterization with Fourier Transform Infrared (FTIR, Bruker, Billerica, MA, USA), Nuclear Magnetic Resonance (NMR, Varian Inc., Palo Alto, CA, USA), gel permeation chromatography (GPC, Malvern Pananalytical Inc., Westborough, MA, USA), and elemental analysis (Elementar, Langenselbold, Germany). This sample was used as a dispersant for kaolin suspensions.

### 2.5. Sulfomethylation

The sulfomethylation of kraft lignin was conducted as previously described for the sulfomethylation of softwood kraft lignin [25]. The sulfomethylation reaction was carried out in a 100 mL three-neck round-bottom glass flask under constant stirring at 150 rpm. A solution of lignin (50 g/L) was prepared under alkaline conditions (pH 11.5–12) using NaOH. After adding Na_2_S_2_O_5_ and CH_2_O, the reaction was carried out for 3 h at 100 °C [25]. The molar ratio of CH_2_O/KL was 1 mol/mol, but the ratio of Na_2_S_2_O_5_/KL was varied (0.5–1.25 mol/mol). The reaction conducted under the conditions of the CH_2_O/KL ratio of 2 mol/mol and the Na_2_S_2_O_5_/KL ratio of 1 mol/mol provided a sample with the highest charge density. This sample (SML) was used for further characterization with FTIR, NMR spectroscopy, gel permeation chromatography (GPC), elemental analysis, and as a dispersant for kaolin suspensions.

### 2.6. Charge Density Analysis

To measure the charge density of lignin derivatives, SBL and SML were dried at 105 °C in an oven overnight. The solution containing 0.2 g of lignin samples and 20 mL of deionized water was prepared and incubated at 30 °C for 1 h in a water bath shaker at 150 rpm. The lignin samples were completely soluble in water. The charge density of lignin samples was measured using a Particle Charge Detector, Mutek, PCD-04 (BTG Instruments, Wessling, Germany). First, 1 mL of a lignin solution and 10 mL of deionized water were added to the PCD titrator cell and titrated against a PDADMAC standard solution (0.005 M). The specific charge density of the samples was determined as explained in previous work [25].

### 2.7. Sulfonate and Phenolate Group Analysis

The sulfonate and phenolate groups of KL, SBL, and SML samples were determined using an automatic potentiometric titrator (Metrohm, Titrando 905, Herisau, Switzerland) via the procedure described previously [25,32]. For the sulfonate group analysis, a solution of the lignin sample was prepared by adding 1 g sample to 100 mL of distilled water, and the pH of the solution was adjusted to 3.0 using 1 M H_2_SO_4_ solution. The sulfonate group content was determined by the potentiometric titration with 0.1 M NaOH solution as the titrant. For the phenolate group analysis, 0.06 g of samples were dissolved in 99 mL of deionized water in a titration beaker. Afterward, 1 mL of potassium hydroxide solution (0.8 mol/L) and 4 mL of para-hydroxybenzoic acid solution (0.5 wt.%) were added to the beaker, and the solution was stirred at 200 rpm for 5 min. Then, the solution was titrated with a 0.1 M hydrochloric acid solution [32].

### 2.8. Molecular Weight Analysis

The SBL and SML samples with a 5 g/L concentration in 0.1 mol/L NaNO_3_ were prepared and stirred at 300 rpm for 24 h. The samples were filtered with a 0.2 µm nylon filter (13 mm diameter), and the filtered solutions were used for the molecular weight analysis. The molecular weight of samples was measured using a gel permeation chromatography, Malvern GPCmax VE2001 Module+ Viscotek TDA305 with multi-detectors (Malvern Pananalytical Inc., Westborough, MA, USA). The flow rate was set at 0.70 mL/min, while the column temperature was 35 °C and poly (ethylene oxide) was used as a standard sample for this aqueous system.

### 2.9. Elemental Analysis

The lignin samples were dried at 105 °C in an oven overnight and the elemental analysis of the dried samples was performed using an Elementar Vario EL Cube elemental analyzer (Elementar, Langenselbold, Germany) by the combustion analysis method. Approximately 2 mg of the lignin sample was burned at 1200 °C in a combustion tube located in the instrument. Afterward, the combustion gases were reduced and analyzed for carbon, hydrogen, nitrogen, and oxygen contents of the samples.

The degree of substitution (DS, mol/mol) of OH of lignin with sulfoalkylate groups was determined based on the sulfur content of the samples according to the Equation (1) [32]:

(1) $$DS=\frac{180\times \left(S_{SL}-S_{KL}\right)}{32-M_{w}\times \left(S_{SL}-S_{KL}\right)}$$

where 180 (g/mol) was considered as the molecular weight of lignin, 32 (g/mol) was the molecular weight of the sulfur element, S_SL_ was the sulfur content of SML or SBL, S_KL_ was the sulfur content of KL, M_w_ was the molecular weight of grafted groups onto lignin (M_w_ = 117 g/mol for SML, and M_w_ = 318 g/mol for SBL).

### 2.10. FTIR Analysis

The FTIR analysis of KL, SBL, and SML were carried out at room temperature using a TENSOR 37 FTIR Spectrophotometer (Bruker, Billerica, MA, USA), which was equipped with a Universal Attenuated Total Reflectance (ATR) sampling (ZnSe cell) and diamond window (about 1.5 mm diameter). Approximately, 0.05 g of oven-dried samples were placed directly onto the ATR crystal using the micrometer pressure clamp. The spectra of samples were recorded at a resolution of 4 cm^−1^ in the range of 600 cm^−1^ to 4000 cm^−1^.

### 2.11. ^1^H– and ^1^H–^1^H COSY NMR Analyses

The structures of the KL, SBL, and SML were analyzed via ^1^H–and ^1^H–^1^H COSY NMR analyses. All signals were referenced to the internal standard, i.e., trimethylsilylpropanoic acid (TSP), at d = 0.00 ppm chemical shift. In this experiment, 40 mg of KL was dissolved in 500 µL of [D_6_]DMSO that contained 2 mg of TSP at 50 °C and shaken in a water bath shaker overnight at 50 rpm. In the same way, 40 mg of SBL and SML were dissolved in 500 µL of 9/1 *v*/*v* [D_6_]DMSO/D_2_O, and the solutions were stirred at 25 °C and 200 rpm overnight to dissolve the material fully. The NMR spectra of all samples were recorded using an INOVA-500 MHz instrument (Varian Inc., Palo Alto, CA, USA). The ^1^H–NMR spectra of KL, SML, and SBL were acquired with a quantity of 16 scans in 128 increments with a 1.0 s relaxation time delay. For the ^1^H–NMR analysis, a 45° pulse, a 4.6 μs pulse width, and a 2.05 s acquisition time were considered. For the ^1^H–^1^H COSY analysis, the acquisition time of 3.983 s, and 16 scans in 128 increments with 1s relaxation time delay were taken into account.

### 2.12. Zeta Potential Analysis

The zeta potential of kaolin suspensions was determined using a NanoBrook Zeta PALS (Brookhaven Instruments Corp, Holtsville, Newyork, USA). In this study, different dosages (0.1–1.0 mg/g based on kaolin) of SML and SBL were added to 50 mL of kaolin suspension (100 g/L concentration) at pH 4.2, 7.8, and 11.3 and stirred at 300 rpm and 25 °C for 1 h. After mixing, their zeta potential was measured in a 1.0 mM KCl aqueous solution. All the measurements were carried out at room temperature with a constant electric field (8.4 V/cm). The experiments were carried out three times and the average values were reported in this study.

### 2.13. Dispersion Analysis under Static Conditions

The stability of the kaolin suspension in the presence of KL, SML, and SBL was investigated using a Turbiscan Lab Expert, Formulaction, Toulouse, France. In this set of experiments, kaolin suspensions (100 g/L) were prepared in the presence of KL, SML, and SBL at varying concentrations (0.1–1.0 mg/g kaolin) by mixing in a water bath shaker at 150 rpm and 30 °C for 1 h. The changes in the stability of the suspensions were analyzed for 2 h (single scans were collected every 2 s) by the instrument at 30 °C. Based on the obtained data, the destabilization index of kaolin dispersion was determined using Turbisoft 2.1 software (Formulaction, Toulouse, France) [31].

### 2.14. Contact Angle Measurement

The wettability of glass slides coated with lignin derivatives was determined by an optical tensiometer instrument (Theta lite, Biolin Scientific, Espoo, Finland). The solutions of the lignin samples (10 g/L) were prepared at pH 7, and 2 mL of sample solution was coated onto microscopic glass slides with a WS-650 spin coater (Laurell Technologies Corp., North Wales, PA, USA) under vacuum with 60 Psi N_2_ pressure at 300 rpm for 30 s. The contact angle of a water droplet (5 µL) on the glass slides coated with lignin samples was determined following the sessile drop method at room temperature.

### 2.15. QCM-D Experiments

A Quartz crystal microbalance with dissipation (QCM-D) was used for studying the adsorption of the SBL and SML on the Al_2_O_3_ coated Quartz sensors (Q-sense Inc., Gothenborg, Sweden) that were used to mimic the positively charged sites of kaolin particles as the results of the mineralogical analysis revealed that kaolin is mainly composed of kaolinite mineral (72.22 wt.%) and contains a high amount of Al_2_O_3_ (43.70 wt.%) (Table S1 in the supporting information file). The buffer solution (Milli-Q water) and lignin solutions prepared in Milli-Q water had the same pH of 7.8–8, at which Al_2_O_3_ substrates were known to be positively charged as they have the isoelectric point of 8.7 ± 0.4 [33].

The adsorption of SBL and SML on Al_2_O_3_–coated crystal sensors was studied using a QCM-D 401, E1, (Q-Sense Inc. Gothenborg, Sweden) at 22 °C. The lignin solutions with different concentrations were prepared in Milli-Q water at pH 7.8–8. The adsorption experiments were started by running the buffer solution as the background solution at a constant flow rate of 0.15 mL/min. Afterward, lignin solutions were passed through the sensor surfaces with the same flow rate of 0.15 mL/min for approximately 25 min until reaching the equilibrium.

Then, the experiments were switched back to the buffer solution for another 5 min to separate unadsorbed lignin polymers from the surface of the sensor.

The changes in the frequency (∆f) and dissipation (∆D) of the sensors were measured simultaneously at 5, 15, 25, 35, 45, 55, and 75 mMHz. The amount of adsorbed mass, Δm (ng/cm^2^), of lignin samples on the surface of the sensors was calculated by using the Sauerbrey’s Equation (2) that is suitable for rigid films adsorbed on the surface using Q-Tools software (Q-sense Inc., Gothenborg, Sweden):

(2) $$∆m=-C\frac{∆f}{n}$$

where ∆m is mass change, ∆f is the frequency change, n is the overtone number and C is the mass sensitivity constant (C = 17.7 ng/cm^2^ Hz at 5 MHz) [34]. The adsorbed layer thickness, d (nm), was calculated following the Equation (3):

(3) $$d=-\frac{∆m}{\rho }$$

where ρ is the assumed density of the polymer layer (ρ = 1.15 g/cm^3^) [35]. The dissipation energy of the sensor (D) indicates the rigidity or softness of the adsorbed layer on the sensor. If the attached layer is viscoelastic, it causes a faster oscillation-damping and as a result, a higher ∆D is observed [36]. In this study, the low values of ∆D < 2 × 10^−6^ indicated the formation of a rigid film, which justified the incorporation of the Sauerbrey Equation (3) in the adsorbed mass analysis. For all calculations, the fifth overtone was used.

## 3. Results and Discussions

### 3.1. Sulfobutylation and Sulfomethylation Reaction Schemes

In this study, two different methods of sulfonation were conducted on lignin. The reaction schemes of sulfobutylation and sulfomethylation of lignin are shown in Figure 1. In the sulfobutylation reaction, the sulfonation reagent, 1,4–butanesultone, reacts with both phenolic and aliphatic hydroxy groups of lignin through S_N_2 mechanism via ring-opening providing a sulfonated group and long alkyl chains (−C_4_H_8_−SO_3_H) grafting to lignin under alkaline conditions [21,26,37].

In the sulfomethylation reaction, sodium metabisulfite (Na_2_S_2_O_5_) acts as the sulfonic acid group provider for lignin, and formaldehyde provides a methyl group for the sulfomethylation reaction [25]. Firstly, a sodium sulfonate methyl derivative is formed by the nucleophilic addition of the sodium sulfite anion to formaldehyde. Sulfomethylation occurs at the ortho position of the aromatic ring of lignin by the electrophilic substitution of the sodium sulfonate methyl derivative [25].

Because the charge density of the SBL and SML is a direct indicator of the extent of sulfoalkylation reaction and a crucial factor in the performance of SBL and SML as dispersants, the effects of reaction conditions on the charge density of lignin derivatives were investigated.

### 3.2. Sulfobutylation and Sulfomethylation of Lignin

**Figure 2:**
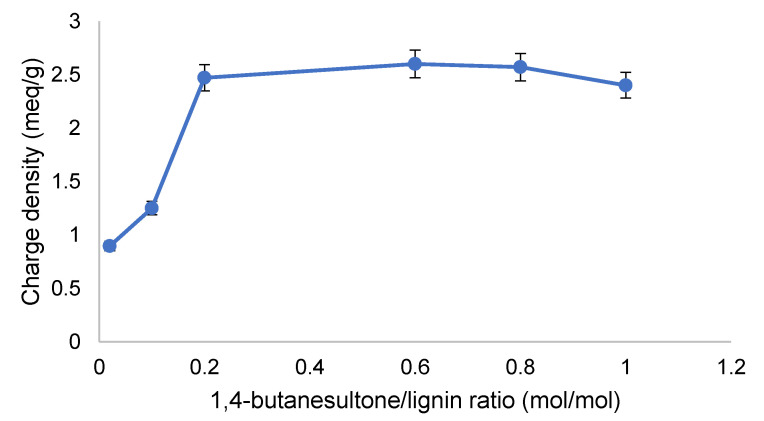
Effect of 1,4–butanesultone to lignin molar ratio on the charge density of sulfobutylated kraft lignin.

The effect of the molar ratio of 1,4–butanesultone/lignin (mol/mol) on the charge density of SBL was investigated under the reaction conditions of 70 °C and 3 h (Figure 2). According to the results, the anionic charge density reached its maximum value at the ratio of 0.2 mol/mol as 1,4– butanesultone is known to be an effective reagent to react with both phenolic and aliphatic groups [21,37,38]. Therefore, the molar ratio of 0.2 mol/mol was selected for the sulfobutylation reaction to produce sulfobutylated lignin.

To produce sulfomethylated lignin with a charge density that was close to that of SBL, different experiments with different ratios of reactants were conducted under the conditions stated in Section 2.5. The charge density of sulfomethylated lignin samples is presented in Table S2 in the supporting information file. As the reactivity of this reaction was rather low [19,25], the molar ratios of the reactants to lignin were increased to obtain a final product with a high degree of sulfonation. The sample produced with formaldehyde to lignin ratio of 2:1 and sodium metabisulfite to lignin molar ratio of 1:1 possessed the highest charge density (−2.31 meq/g), which was close to that of sulfobutylated lignin (−2.37 meq/g) and this sample was selected for further assessment.

### 3.3. FTIR Analysis

The FTIR spectra of KL, SBL, and SML are provided in Figure S1 in the supporting information file. In general, the broadband at 3404 cm^−1^, which was attributed to the stretching vibration of –OH groups and hydrogen bonds [39], was smaller for SBL and SML than KL, revealing that hydroxy groups of KL were bound to other groups after modification reactions. The relative intensity at 1600 cm^−1^ was smaller in the SML spectrum than in the KL spectrum, which was the evidence for the aromatic cleavage, as that peak would characterize the aromatic functionality of lignin [40]. SBL had a weak absorbant peak of the phenolic group (stretching at 3404 cm^−1^), which indicated that alkyl groups were bound to the phenolic hydroxy groups. The vibrations at 1202 cm^−1^ were attributed to the phenyl C–O–C ether bond stretching, which indicated the introduction of alkyl chain to lignin via sulfobutylation [21]. The signal at 1080 cm^−1^ refers to the symmetric stretching vibration of SO_3_H group [41]. Thus, these results reveal that the ring-opening of 1,4–butane–sultone occurred and the sulfonate group was successfully anchored onto the KL.

### 3.4. H–NMR Spectroscopy and ^1^H–^1^H COSY Spectroscopy

**Figure 3:**
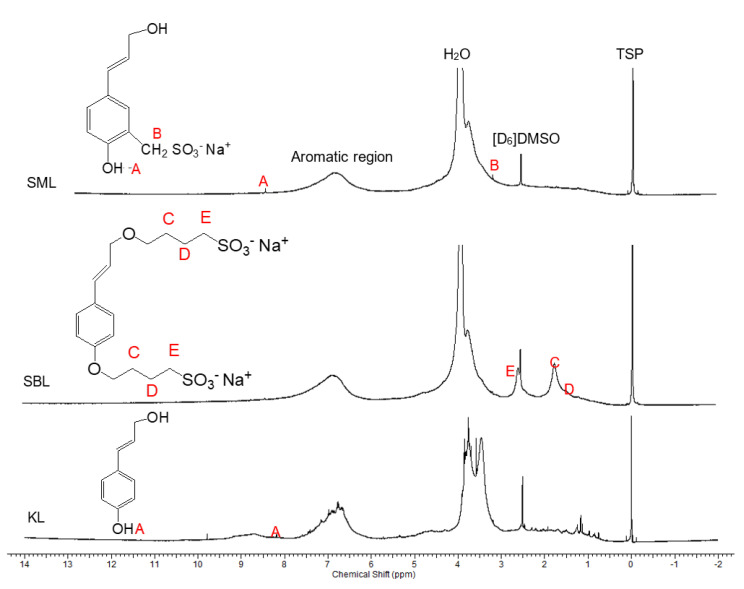
^1^H–NMR Spectra of kraft lignin (KL), sulfomethylated lignin (SML), and sulfobutylated lignin (SBL) in [D_6_]DMSO.

The ^1^H–NMR spectra of KL, SML, and SBL in [D_6_]DMSO were presented for the qualification of the structures of the lignin derivatives in Figure 3. In all spectra, protons in water appear at 3.95 ppm, and the peak at 2.58 ppm is related to the protons of [D_6_]DMSO. In the spectrum of KL, two broad peaks at 6.8–7.5 ppm and 3.2–3.8 ppm represent the protons of the aromatic ring and methoxy groups, respectively [39]. In the spectrum of SML, these peaks are weaker than in that of KL due to the breakage of aromatic ring and methoxy groups during sulfomethylation reactions [25]. The peak at 8.2 ppm (shown as proton A) represents the hydrogen of the phenolic hydroxy, which is present in the spectra of the KL and SML, implying that phenolic hydroxyl groups in lignin remained unaffected during sulfomethylation reactions. In SML’s spectrum, the peak at 3.23 ppm (assigned as proton B) is the signal of –CH_2_ groups in sulfomethylated lignin [42]. In the spectrum of SBL, three peaks at 1.79, 1.58 and 2.63 ppm can be assigned to C, D, and E protons (Figure 3), respectively, which are not present in the spectrum of KL. The remarkable increase in these peaks is related to the aliphatic proton signals arising from the coupling of the −C_4_H_8_−SO_3_H groups, and they proved the production of sulfobutylated lignin [21,26,37].

**Figure 4:**
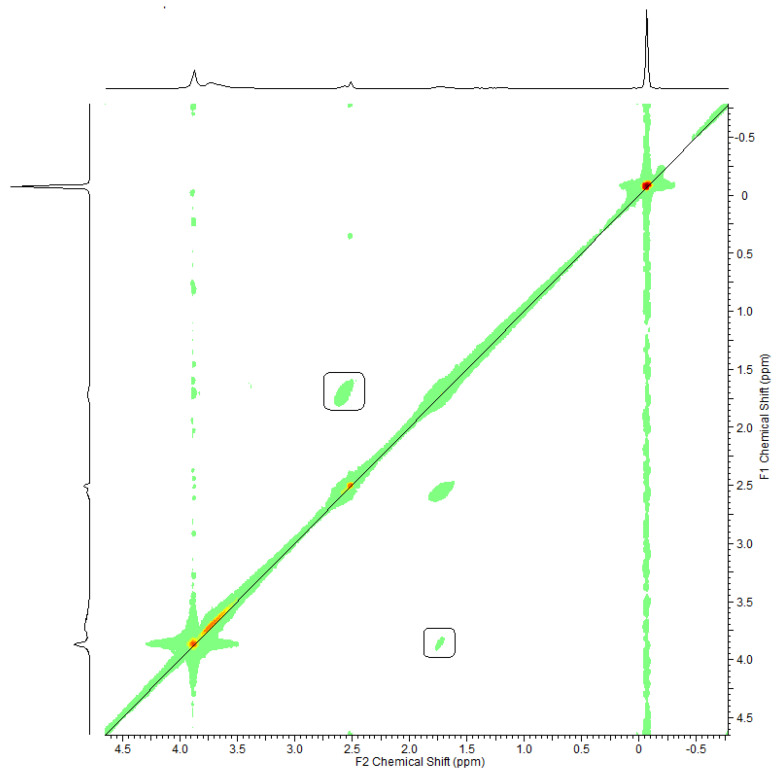
^1^H–^1^H 2D COSY spectrum of SBL. The coupled protons are shown in the squares.

In this work, ^1^H–^1^H COSY spectroscopy was used to reveal the hydrogens in the alkyl chain (–C_4_H_8_–) that was attached to the lignin structure via the sulfobutylation reaction. The schematic COSY spectrum of SBL was presented in Figure 4. The two cross-peaks were observed at F1 = 1.79 ppm and F2 = 2.63 ppm as well as F1 = 2.63 ppm and F2 = 1.79 ppm implying that these hydrogens were coupled. Moreover, the peaks at F1 = 3.86 ppm and F2 = 1.79 ppm as well as at F1 = 1.79 ppm and F2 = 3.86 ppm resemble the positions of other protons that are coupled. The information from ^1^H–^1^H COSY spectroscopy provides evidence for the existence of butyl group in the lignin structure. In the ^1^H–^1^H COSY spectra of KL and SML (Figures S2 and S3 in the supporting information files, respectively), no signals were observed indicating the existence of coupled protons as these structures had no alkyl chains attached to the aromatic ring.

### 3.5. Properties of KL, SBL, and SML

The properties of KL, SML, and SBL are tabulated in Table 1. Generally, there was an increase in the sulfonate group content for SML and SBL compared with KL (1.88 and 1.89 vs 0.02 mmol/g, respectively), which reflects the success of sulfonation of KL. As the amount of sulfonate group attached to KL was the same for both reactions, the degree of substitution values was observed to be the same (0.22). Their similar sulfonate group is associated with their similar charge density. Both lignin derivatives possessed higher sulfur and oxygen elements than KL, which is another evidence for the sulfonation of KL. The decrease in the amount of phenolic hydroxy groups of KL after sulfobutylation provides evidence for not participation of these groups in the sulfobutylation reaction (Table 1). On the other hand, the number of phenolic hydroxy groups of KL remained unchanged after sulfomethylation (Table 1), indicating not participating of the phenolic hydroxy groups in the sulfomethylation reaction. This finding is in agreement with the results of ^1^H–NMR discussed above (Figure 3). The molecular weight of KL (17,890 g/mol) was increased to 34,890 g/mol after sulfobutylation, which is attributed to the grafting of 1,4–butane sultone onto KL [26]. Sulfomethylation reaction also increased the molecular weight of KL, but to a smaller degree than SBL. The main reason for the larger molecular weight of SBL is the addition of a longer carbon chain, butyl group (–C_4_H_9_) than methyl group (–CH_3_), to KL and the attachment of this group to both aliphatic and aromatic groups (compared with only one group to the aromatic group in sulfomethylation reaction). The polydispersity of SML was smaller than KL, implying that the molecular weight distribution of KL was improved and lignin became more homogeneous by the sulfomethylation process. However, polydispersity insignificantly changed for SBL, indicating that sulfomethylation increased the molecular weight of lignin with a more uniform distribution than sulfobutylation did.

### 3.6. Adsorption Analysis of Lignin Derivatives on Aluminum Oxide Coated Surfaces

**Figure 5:**
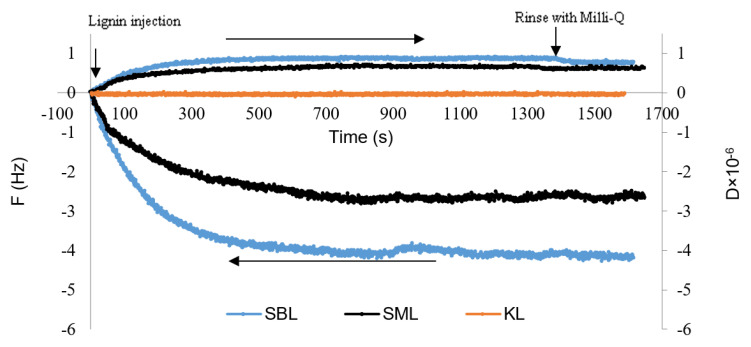
Adsorption of lignin derivatives (500 mg/L concentration) on Al_2_O_3_ coated Quartz sensors at pH 7.8.

The adsorption of KL, SBL, and SML onto the Al_2_O_3_ coated Quartz sensors for six different concentrations (100–600 mg/L) were conducted to determine their adsorption performance on the surface at pH 7.8. The frequency and dissipation of the Quartz sensor for the adsorption of lignin derivatives at 500 mg/L concentration are given in Figure 5. As seen, there was no change in frequency and dissipation for KL, which means that KL did not adsorb on the surface of the sensor. However, a significant drop in the frequency was observed for SBL and SML, implying their adsorption on the sensor (Figure 5), and the change was more pronounced for SBL.

With the continued injection of lignin solutions, frequency values decreased gradually until they reach an equilibrium state at approximately −4.18 Hz and −2.69 Hz for the SBL and SML, respectively. While frequency values dropped, dissipation values remained stable at values under 2 × 10^−6^ (0.77 × 10^−6^ and 0.62 × 10^−6^ for SBL and SML, respectively), which suggested the formation of rigid adlayers as time elapsed [43].

The plots of frequency and dissipation for other concentrations are presented in Figure S4 in the supporting information file. Similarly, the formation of a rigid adlayer of SBL and SML was observed for all concentrations with dissipation values under 2 × 10^−6^ (Figure S5).

**Figure 6:**
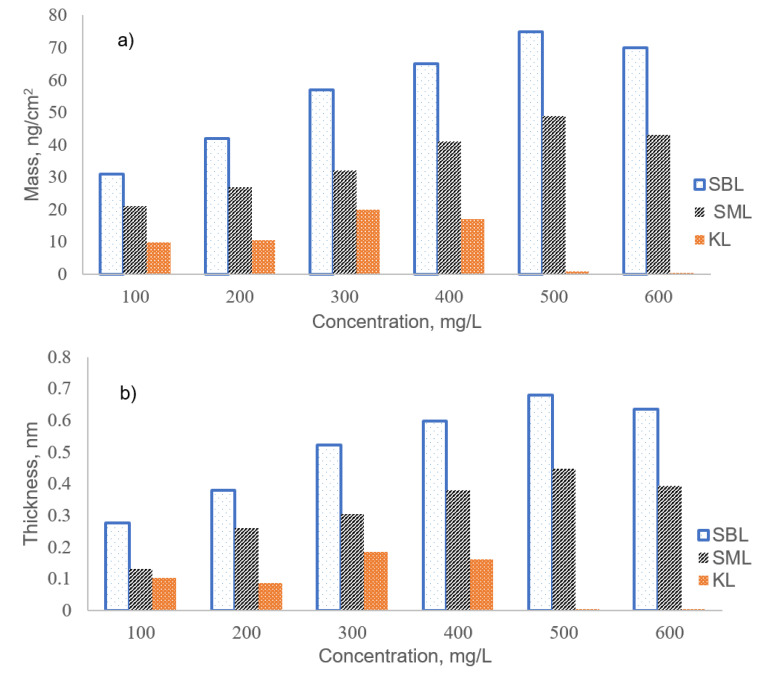
Adsorption of KL, SBL, and SML at different concentrations (**a**) Mass uptake (**b**) Thickness.

Figure 6 shows the final adsorbed mass and thickness determined from the changes in the frequency using the Sauerbrey Equation for different concentrations of lignin solutions. KL had the lowest adsorbed mass and thickness at all concentrations when compared with SBL and SML, implying the limited affinity of KL for adsorption on the surface at pH 7.8. For all concentrations, the adsorbed mass and thickness for SBL were higher than SML confirming that the higher molecular weight lignin derivative adsorbed more than the lower molecular weight one on the surface [44,45]. The maximum mass uptakes for both SBL and SML (74.71 ng/cm^2^ and 49.54 ng/cm^2^, respectively) were observed at the concentration of 500 mg/L, and this concentration was considered as the saturation concentration for the adsorption of both lignin derivatives onto the Al_2_O_3_ surface. Moreover, the higher *M*_w_ and longer and softer alkyl chains of SBL than SML caused a better anchoring effect [26] (Figure 1) resulting in higher mass uptake of SBL on the surface (Figure 6a). However, increasing the concentration to 600 mg/L dropped the adsorbed mass of all lignin derivatives. This behavior is attributed to the competitive occupancy and the repulsive interaction between the excess molecules [46] that could hinder the adsorption.

The contact angle of water with KL, SML, and SBL coated surfaces was 23.1° ± 0.3, 6.3° ± 0.2, 8.7° ± 0.5, respectively, implying the more hydrophilic nature of lignin derivatives due to the grafting of sulfonate functional groups. SBL has a slightly higher hydrophobic character than SML due to having longer alkyl chains that could lead to a higher tendency of SBL molecules to stay attached to the surface. According to the literature, the contact angle of kraft lignin can be larger if lignin is protonated in an acidic solution rather than deprotonated in an alkaline solution. Thus, the rather small contact angle of KL in this study might be related to the presence of Na^+^ and its generation in an alkaline environment that made lignin deprotonated and somehow hydrophilic [30].

#### 3.6.1. Adsorption Kinetics

**Figure 7:**
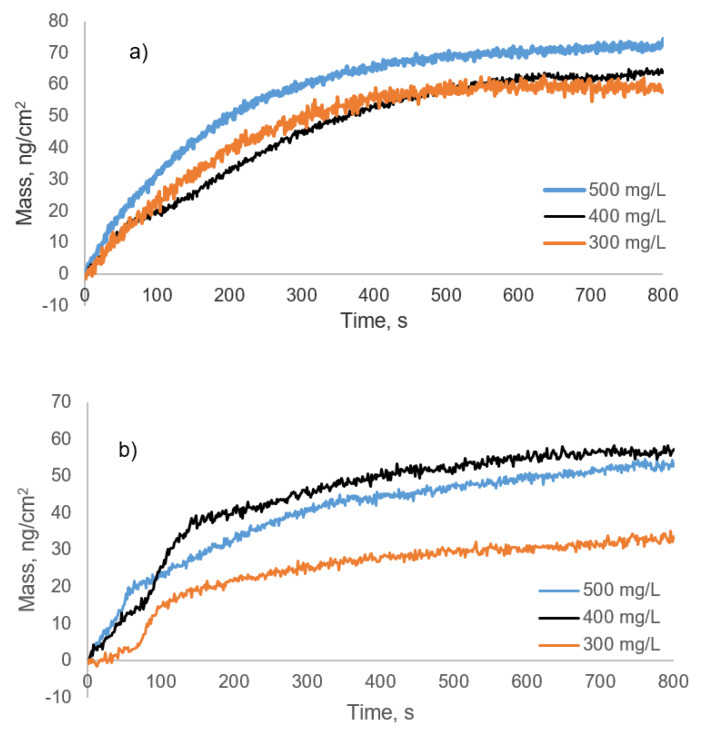
Kinetics of the mass deposition on the Al_2_O_3_ surface for the adsorption of (**a**) SBL (**b**) SML.

The QCM-D data allows for the kinetic analysis of the adsorption profile for lignin derivatives. The kinetics of mass deposition on the Al_2_O_3_ surface for lignin solutions for the concentrations of 300, 400, and 500 mg/L were presented in Figure 7. For KL, the kinetics of mass deposition is presented in Figure S6 in the supporting information files file. The data points of adsorption were fitted into a pseudo-first-order rate equation (Equation (4):

(4) $$\Gamma \left(t\right)=\Gamma_{e}\left(1-e^{-\mathrm{kt}}\right)$$

where Γ(t) is the adsorbed mass (ng/cm^2^) at time t (seconds), k is the apparent rate constant of adsorption (1/s), and Γ_e_ is the adsorbed mass at equilibrium. Fitting parameters and correlation coefficients for the lignin adsorption are presented in Table 2. For lignin derivatives, reasonable correlations were observed for the fitting, and the fitted values (Γ_e_); and the experimental values (Γ_exp_) of the adsorbed mass were in good agreement (Table 2). On the other hand, the limited adsorption of KL hampered the adsorption (Table S3 in the supporting information file). The adsorption kinetics of lignin derivatives have two distinct stages: the first one is a rapid initial uptake of the lignin polymer, governed by the mass transport rate, and a second stage is a slow approach to adsorption equilibrium, which may take longer times than the first stage [47]. If the polymer adsorption is controlled by the second step (attachment step), it has a kinetic profile that could be fitted into a pseudo-first-order expression [48]. The adsorption kinetics of SBL and SML indicated a first-order exponential growth relationship between adsorbed amount and time, implying that the rate-determining step in the adsorption of the lignin derivatives was the adsorption step [48,49]. Deviation from pseudo-first-order kinetics for polymer adsorption indicates the existence of a conformational rearrangement of macromolecules on the surface before adsorbing more polymers. This is because the conformation change could limit the extensive adsorption, with adsorption at later times relying on the rearrangement of polymer chains already adsorbed on the surface [49,50].

#### 3.6.2. Adsorbed Layer Characteristics

**Figure 8:**
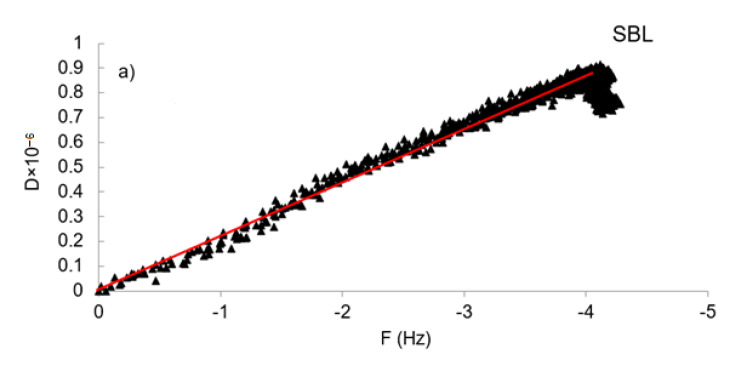

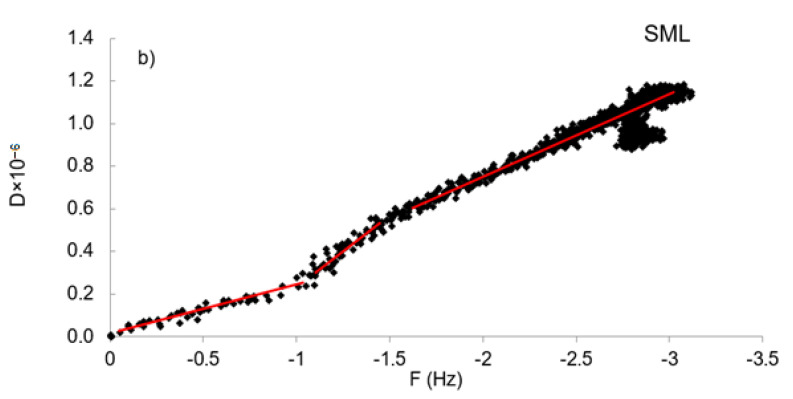
Dissipation change of the sensors as a function of frequency change for (**a**) SBL (**b**) SML for 500 mg/L concentration.

The changes in the slopes of ΔD/ΔF plots were investigated and presented in Figure 8 for the concentration of 500 mg/L, and for concentrations of 300 and 400 mg/L in Figure S7 in the supporting information file. The absolute values for the slopes of ΔD/ΔF plots were presented as K values in Table S4 in the supporting information file for making quantitative distinctions for the properties of adsorbed layers. The plot for SBL (Figure 8a) was linear during the whole adsorption process, which indicates that no conformational rearrangement was observed for the SBL molecules as more mass adsorbed on the surface [43]. For SML, at the first stage of adsorption, the plot started with a small K value indicating a rigid adlayer (Figure 8b; Table S4), but the slope of the second region (K_2_) was considerably steeper than the initial slope, signaling more dissipation per adsorbed molecule [46]. Possibly, conformational changes and water adsorption alter the flexibility of the adsorbed layer, and the change of dissipation arises [51]. The observation of conformational rearrangement for SML is in good agreement with the kinetic data (Table 2), as the reorientation step causes a deviation from pseudo-first-order kinetics. The slope in the reorientation step of SML was steeper than the slope for SBL, illustrating that the molecules of SML exhibited a more extended configuration from the surface forming a more irregular layered film [35], presumably due to higher hydrophilicity of SML. In the saturation region of SML adsorption, a smaller slope, K_3_, was observed (Table S4), due to the departure of entrapped water molecules from the adlayer by adsorbed polymer molecules as the adsorption continued leading to a more compact film on the surface [44].

### 3.7. Zeta Potential Analysis

**Figure 9:**
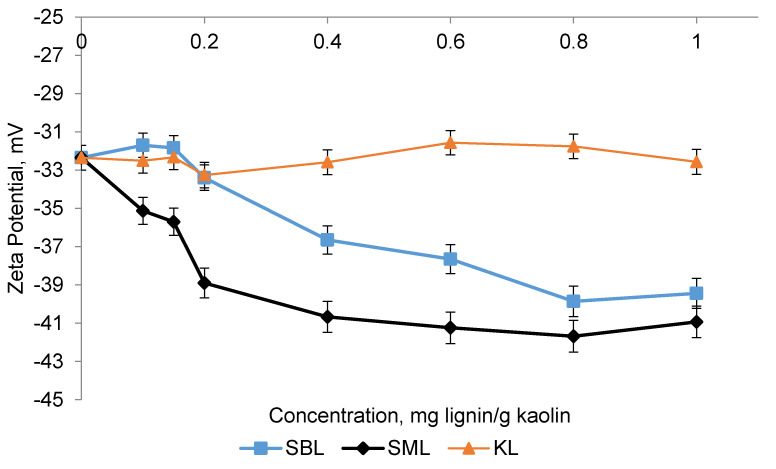
Effect of lignin derivative dosage on zeta potential.

The zeta potential of kaolin particles is an important parameter of suspensions as it represents the strength of the electrostatic repulsive interaction between particles [8]. The kaolin particles with the surface area of 24.16 m^2^/g and a particle size of 7.96 μm were used in this study. Figure 9 shows the effect of KL, SBL, and SML on the zeta potential of kaolin suspensions at different concentrations. KL insignificantly affected the zeta potential of kaolin suspensions. However, with the addition of sulfoalkylated lignins, the zeta potential of the suspension became more negative [52]. The minimum zeta potential values were obtained at the dosage 0.8 mg/g of kaolin (−40 and −42 mV for the SBL and SML, respectively). It is also apparent that the SML reduced the zeta potential of the suspension more greatly. The difference in the zeta potential values for sulfoalkylated lignins with similar charge densities could stem from the thicknesses of adsorbed layers of sulfoalkylated lignins. As seen from the results in Figure 6b, the total adlayer for SML is thinner than SBL for all concentrations. The thinner adsorbed layer of SML could cause a more negative zeta potential as a thinner electrical double layer favors a negative zeta potential [52]. In another study, an increase in the zeta potential of kaolin suspensions was higher for a linear polymer with a thinner adsorbed layer on a particle [12]. The configuration types of lignin derivatives on the surfaces could have an impact on the negativity of the zeta potential. As discussed in Section 3.6.2., SBL formed a more rigid adlayer, while SML had a more extended configuration on the surface. A more compact configuration on the surface could diminish the number of free chains available to interact with the surface, whereas an extended polymer chain could facilitate the electrostatic interactions between charged groups on the polymer backbone and the surface [53,54]. In a former study, polymers adsorbed on the kaolin surface with a more extended configuration and impacted the value of zeta potential more greatly [55].

### 3.8. Destabilization Analysis

**Figure 10:**
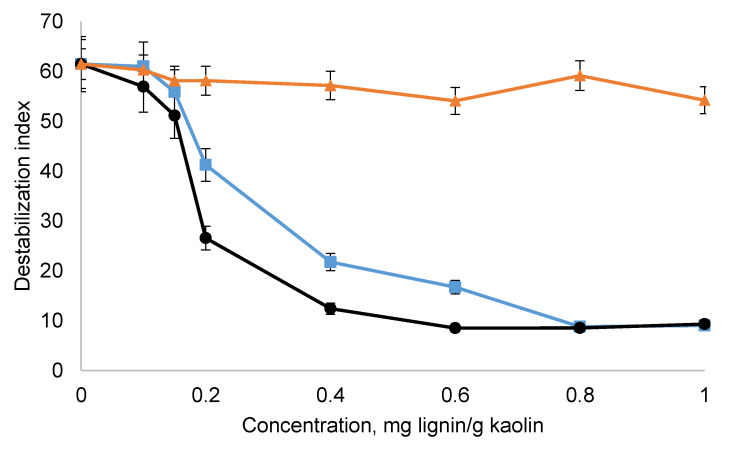
Effect of lignin dosage on destabilization index.

Figure 10 shows the destabilization index of kaolin suspensions in the presence of lignin derivatives. KL could not disperse the suspension, but the other lignin derivatives reduced the instability of the suspension greatly. This improvement in the stability of kaolin suspension is due to the electrostatic repulsion generated between the particles (Figure 9) by the adsorption of lignin derivatives (Figure 6). As SML affected the zeta potential more greatly (Figure 9), it impacted the instability of the suspension more sensibly. It is also apparent that the higher molecular weight of SBL did not promote its dispersion performance. In a previous study, the effect of the molecular weight of oxidized lignin on the performance of kaolin dispersion was investigated [31].

### 3.9. Analysis at Different pH

The adsorption of lignin derivatives on Al_2_O_3_ surfaces, zeta potential, and destabilization of kaolin suspensions at different pH values were investigated. The adsorption studies were conducted at 500 mg/L concentration, as it was found to be the saturation concentration for both lignins (Figure 6). The zeta potential analysis was conducted at a 0.8 mg/g kaolin dosage at which the minimum zeta potential values were obtained (Figure 9). Results showed that (Table 3) the adsorption was higher for both SBL and SML at higher pH. The higher adsorption of lignin derivatives at higher pH was due to the increase in the extent of deprotonation of sulfonic groups to form anionic groups (R–SO_3_^–2^), which caused lignin derivatives to adsorb on the Al_2_O_3_ surface via electrostatic attraction [56,57]. The negativity of zeta potential was higher for both SBL and SML at higher pH, which was due to the increase in the adsorption of lignin derivatives on the positive sites of kaolin particles [6]. In the absence of lignin derivatives, the destabilization index of kaolin particles was lower at higher pH, which is due to the stabilization of kaolin particles as a result of high electrostatic repulsion forces between the kaolin particles at elevated pH, i.e., higher zeta potential (Table 3). KL did not affect the zeta potential and destabilization index of kaolin suspensions at any pH (Table 3). In the presence of lignin derivatives, the kaolin dispersion was improved at higher pH for both SBL and SML (Table 3). As discussed above, the presence of lignin derivatives increased the negativity of the zeta potential and elevated electrostatic repulsion force between particles more greatly at higher pH (Table 3). Similar behavior was observed in another study in the dispersion of kaolin with lignin-acrylamide polymer when the elevated negativity of zeta potential and a higher level of dispersion was observed at pH 10 than other pHs [30]. The results also showed that the lignin derivatives were not sufficiently strong to disperse kaolin particles under acidic pH due to the strong interaction between the positively charged edge surfaces and negatively charged basal surfaces of kaolin particles [58]. However, they were very effective in dispersing the particles at neutral pH, at which the edge surfaces became partially negatively charged, and with increasing pH, kaolin particles attained a state of homogeneity in charge, i.e., having negative at both edge and basal surfaces [59]. As a result, under alkaline conditions, kaolin suspension did not need a dispersant. These results reveal that the effect of the dispersant is switchable with pH, which implies that the dispersion of kaolin suspension can be interrupted by acidifying the suspension. This behavior is advantageous for some applications, such as separation and purification processes, where kaolin particles are in effluent streams and pH change is used for coagulating, thus removing them from the system.

### 3.10. Comparison of SBL and SML

Although sulfomethylation is a widely accepted process for lignin modification to obtain negatively charged lignin derivative, the use of phenol as a solvent in the sulfomethylation reaction is a major drawback of the process, as phenol is toxic. On the other hand, sulfobutylation proceeds in an aqueous medium. Additionally, the amount of required chemical, 1,4–butane sultone, to generate sulfonated lignin with a similar charge density to SML was lower for SBL than for SML implying its advantages in terms of higher production efficiency and limited environmental footprint. The results of this work confirmed that SBL could have similar dispersion performance as SML at the dosage of 0.8 mg/g (Figure 10). At lower dosages, SML had slightly better dispersion performance (approximately 15%). The pH studies confirmed that both SBL and SML stabilized the suspension at neutral pH. In this work, we introduced sulfobutylation as a more environmentally friendly alternative to sulfomethylation for producing a sulfoalkylated lignin for the dispersion of kaolin suspension.

## 4. Conclusions

In this work, two sulfoalkylated lignin derivatives with different alkyl chain lengths were prepared via sulfobutylation and sulfomethylation routes. SBL with additional long alkyl chains had a higher molecular weight and hydrophobicity than SML. The results of adsorption studies conducted via the QCM-D studies revealed that SBL was adsorbed more greatly than SML on the Al_2_O_3_ surface, and thus it generated a thicker but less viscoelastic adlayer on the surface. The adsorption kinetics of SBL and SML on the Al_2_O_3_ surface was fitted into pseudo-first-order kinetic models. However, a deviation from pseudo-first-order kinetics was observed for the SML, which indicated the presence of a conformational rearrangement step in the adsorption process of SML. The tendency of SML to form a more viscoelastic and a thinner adlayer on the surface (compared with SBL) was attributed to the higher hydrophilicity of SML molecules that tended to interact with water more greatly. The SML/kaolin mixture tended to have a more negative zeta potential and lower destabilization index in the concentration range of 0.15–0.6 mg/g SML/kaolin. Both lignin derivatives were effective in dispersing the suspension at neutral pH, and their efficiency was diminished in an acidic environment. The results confirmed that not only the amount of adsorption but also the properties of the adsorbed layer impacted the zeta potential and dispersion performance of sulfoalkylated lignins.

## Figures and Tables

**Table 1:** Properties of KL, SBL, and SML.

Sample	Charge Density meq/g ±0.002	Sulfonic Acid Group Content mmol/g ±0.002	Phenolic Group Content mmol/g ±0.02	Degree of Substitution mol/mol	*M*_w_ g/mol	*M*_n_ g/mol	*M*_w_/*M*_n_	C (wt.%)	H (wt.%)	O (wt.%)	S (wt.%)	N (wt.%)
KL	0.01	0.02	2.65	-	17,890	5150	3.47	64.8	5.8	26.7	0.11	0.03
SBL	−2.37	1.89	1.78	0.22	34,890	11,100	3.14	55.8	5.7	35.1	3.4	0
SML	−2.31	1.88	2.42	0.22	21,950	9270	2.34	53.8	5.3	37.3	3.6	0

**Table 2:** Pseudo-first order fitting parameters for the mass uptake of lignin.

Concentration (mg/L)	Lignin Derivative	R^2^	k (s^−1^)	Γ_e_ (ng/cm^2^)	Γ_exp_ (ng/cm^2^)
300	SBL	0.99	0.0051	62.2	58.2
	SML	0.97	0.0042	34.7	35.8
400	SBL	0.99	0.0034	69.9	64.3
	SML	0.98	0.0057	57.1	56.1
500	SBL	0.99	0.0058	72.7	74.4
	SML	0.98	0.0049	53.4	53.6

**Table 3:** Properties for suspensions at different pH values (500 mg/L concentration and 0.8 mg lignin/g kaolin for zeta potential and destabilization index were used).

	Adsorbed Mass (ng/cm^2^)			Zeta Potential (mV)			Destabilization Index		
	pH 4.2	pH 7.8	pH 11.3	pH 4.2	pH 7.8	pH 11.3	pH 4.2	pH 7.8	pH 11.3
Kaolin	-	-	-	−22.93	−32.35	−41.45	63.36	61.47	8.08
KL	n/a	n/a	n/a	−24.12	−31.76	−40.30	66.92	59.15	11.83
SML	23.93	49.54	52.30	−37.53	−41.68	−43.59	65.03	8.51	7.29
SBL	48.45	74.71	85.40	−34.50	−39.86	−45.83	69.69	8.53	7.79

## Supplementary Materials

The following are available online at https://www.mdpi.com/2073-4360/12/9/2046/s1, Table S1: mineralogical properties of kaolin, Table S2: Charge density of sulfomethylated lignin, Table S3: Pseudo-first-order fitting for KL, Table S4: dissipation coefficent, Figure S1: FTIR spectra of lignin derivatives, Figure S2: H–H COSY NMR of KL, Figure S3: H–H COSY NMR of SML, Figures S4 and S5: kinetics of adsorption of lignin derivatives, Figure S6: mass deposition of KL, Figure S7: dissipation of lignin derivatives upon adsorption.
