# Supplementary material for: Using Sulfobutylated and Sulfomethylated Lignin as Dispersant for Kaolin Suspension
Source: Polymers (Basel). 2020 Sep 8;12(9):2046. doi: 10.3390/polym12092046 (PMC7570282; doi:10.3390/polym12092046)
Supplement: Supplementary file 1 [file polymers-12-02046-s001.pdf]

Supporting information

Using Sulfobutylated and Sulfomethylated Lignin as Dispersant for Kaolin Suspension

Derya Yesim Hopa,[a, b] Pedram Fatehi*[a,c]

[a] Department of Chemical Engineering, Lakehead University, Thunder Bay, ON, Canada P7B 5E1.
[b] Department of Chemical Engineering, Afyon Kocatepe University, Afyonkarahisar, 03200, Turkey.

[c] State Key Laboratory of Biobased Material and Green Papermaking, Qilu University of Technology (Shandong Academy of Sciences), Jinan, China, 250353

The mineralogical analysis of a 0.5 g of air-dried kaolin sample was carried out via X-Ray Diffraction (XRD) analysis using Pananalytical Expert Pro Diffractometer (Malvern, UK) with a Cu Kα (λ = 1.5405 Å) radiation source. The sample was scanned between 6°-95° with a step of 0.02° per second. A phase identification software (Match, Crystal Impact, Bonn, Germany) was used to determine the mineralogical composition of kaolin.

Table S1. The results of mineralogical analysis and calculated oxide composition for kaolin.

Mineral	Weight (%)	Calculated oxides	Weight (%)
Kaolinite	72.22	Al_2O_3	43.70
Dickite	18.53	SiO_2	55.17
Quartz	3.24	Fe_2O_3	0.62
Cordierite	6.02	MgO	0.50

Table S2. Charge density values of sulfomethylated lignin with different ratios of formaldehyde to lignin (F/L) and sodium metabisulfite to lignin (S/L).

F/L = 1:1	S/L	Charge density (meq/g)
	0.5:1	-2.00
	0.75:1	-2.06
	1.25:1	-2.15
F/L = 2:1	1:1	-2.31

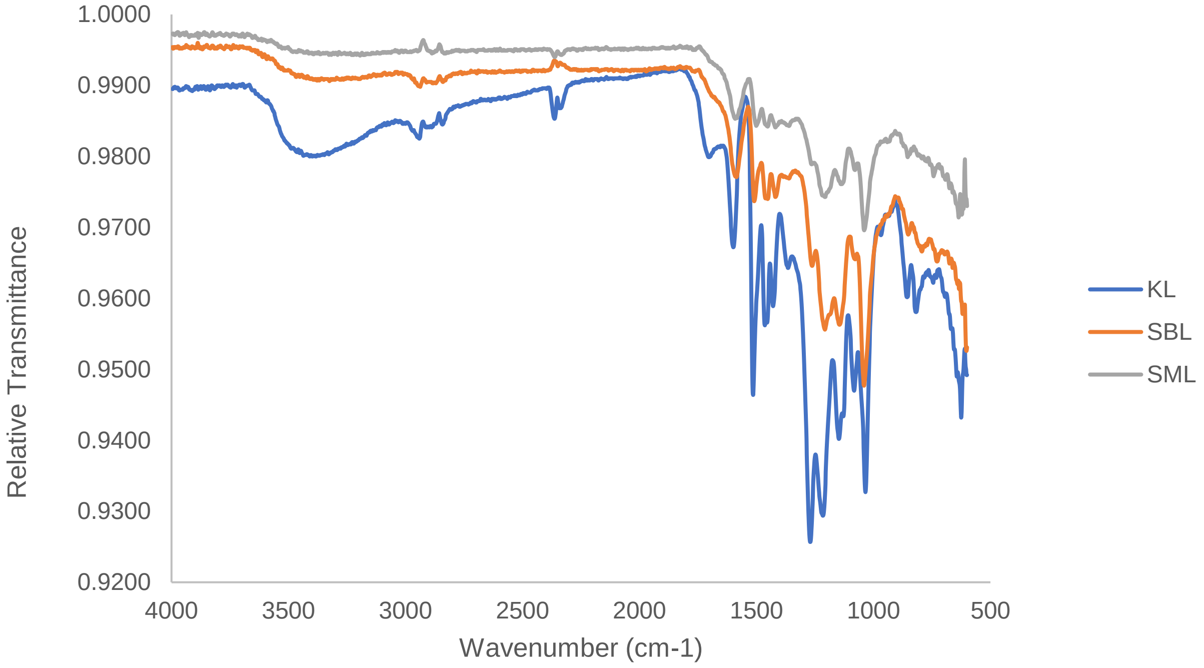

Figure S1. FTIR spectra of modified lignin (SBL and SML) samples compared with KL.

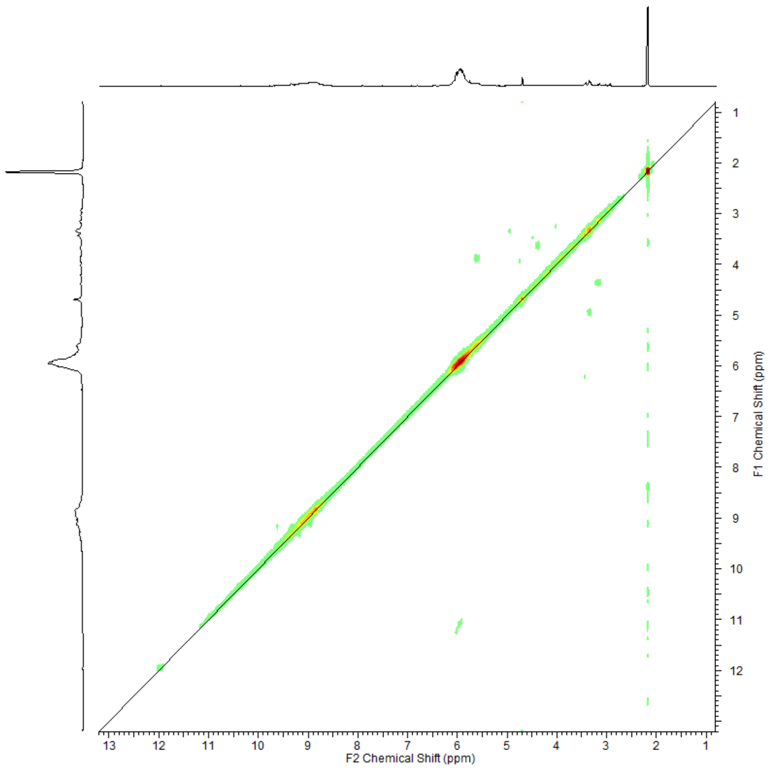

Figure S2. ^{1}H-^{1}H 2D COSY map of KL.

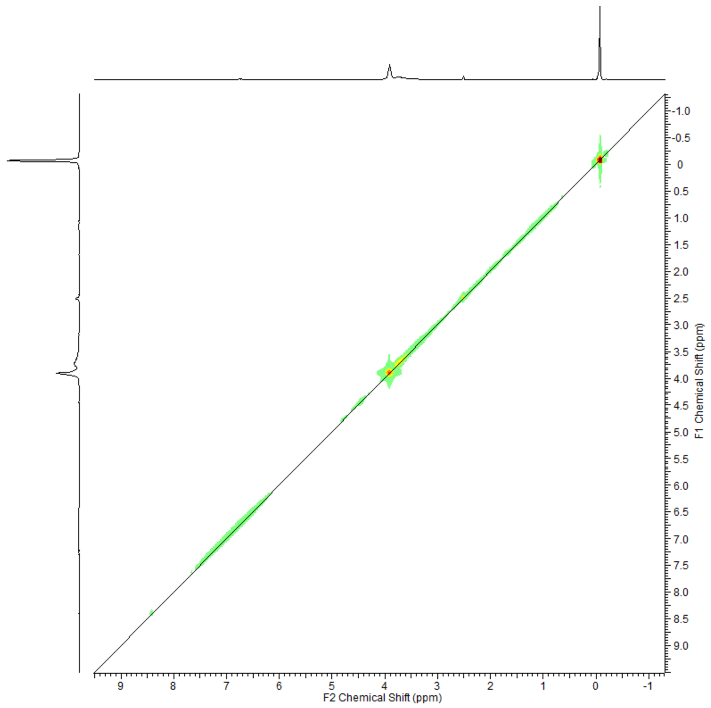

Figure S3. ^{1}H-^{1}H 2D COSY map of SML.

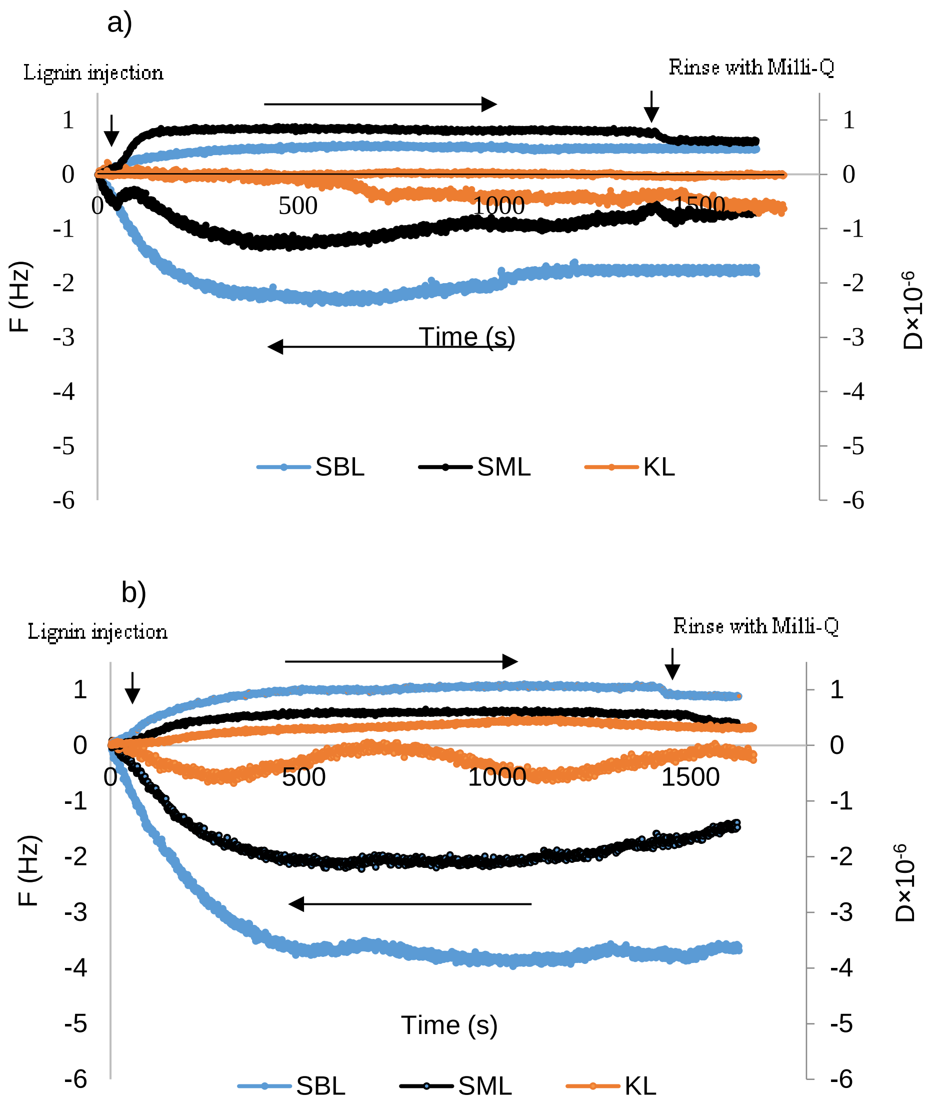

Figure S4. Adsorption of lignin derivatives on Al_2O_3 coated quartz sensors for concentrations **a)** 100 mg/L **b)** 200 mg/L at pH 7.8.

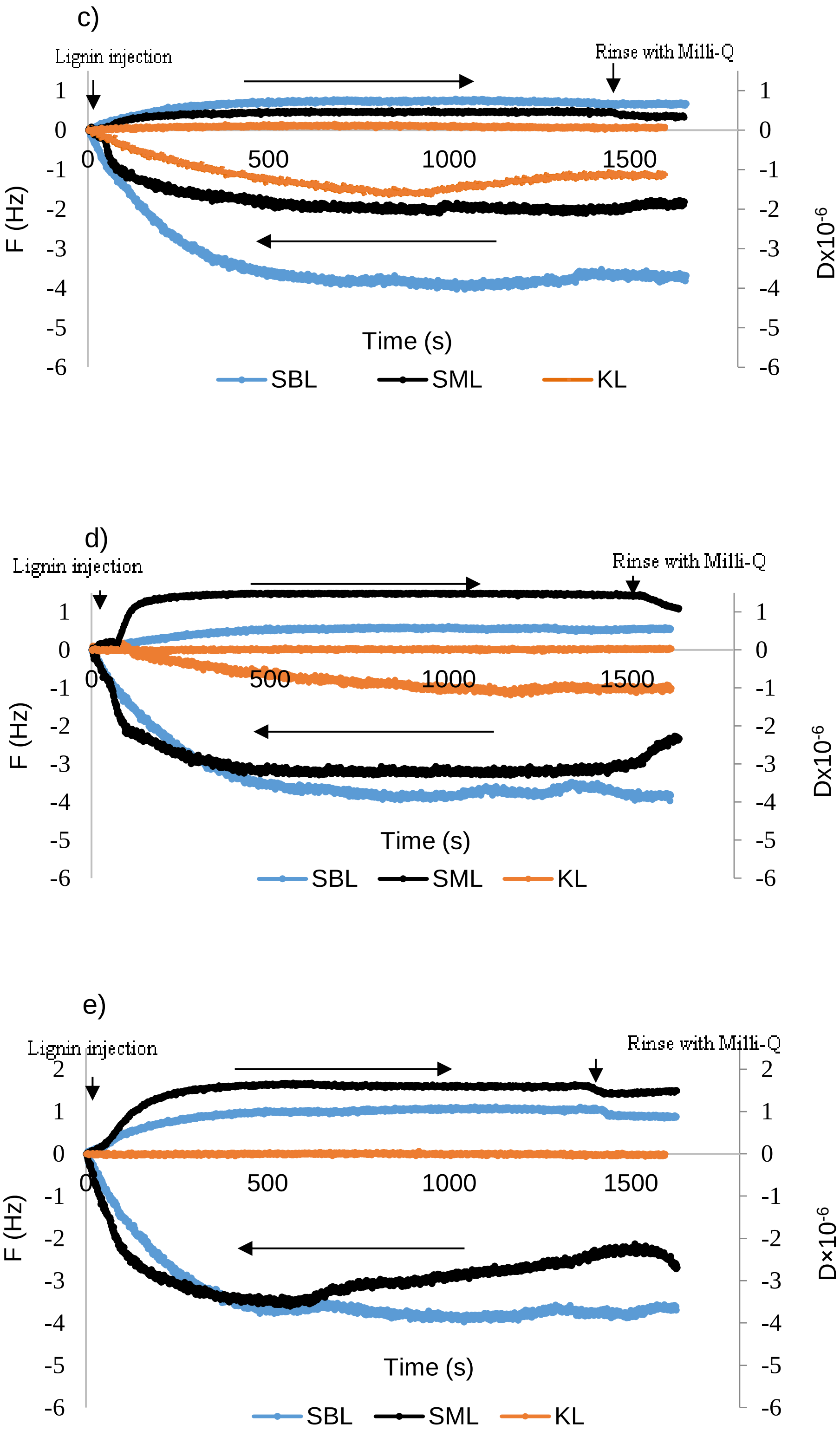

Figure S5. Adsorption of lignin derivatives on Al_2O_3 coated quartz sensors for concentrations **c)** 300 mg/L **d)** 400 mg/L **e)** 600 mg/L at pH 7.8.

Table S3. Pseudo-first-order fitting parameters for the mass uptake of KL

Concentration (mg/L)	R^2	k (s^{-1})	Γ_e (ng/cm^2)	$\Gamma_{exp.}$ (ng/cm^2)
300	0.99	0.0021	32.4	26.8
400	0.93	0.0004	53.7	16.2

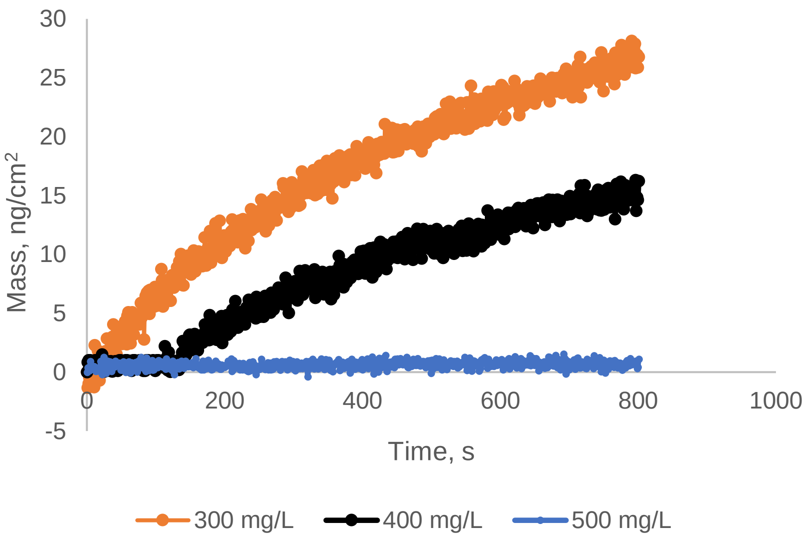

Figure S6. Kinetics of the mass deposition on Al_2O_3 surface for the adsorption of KL

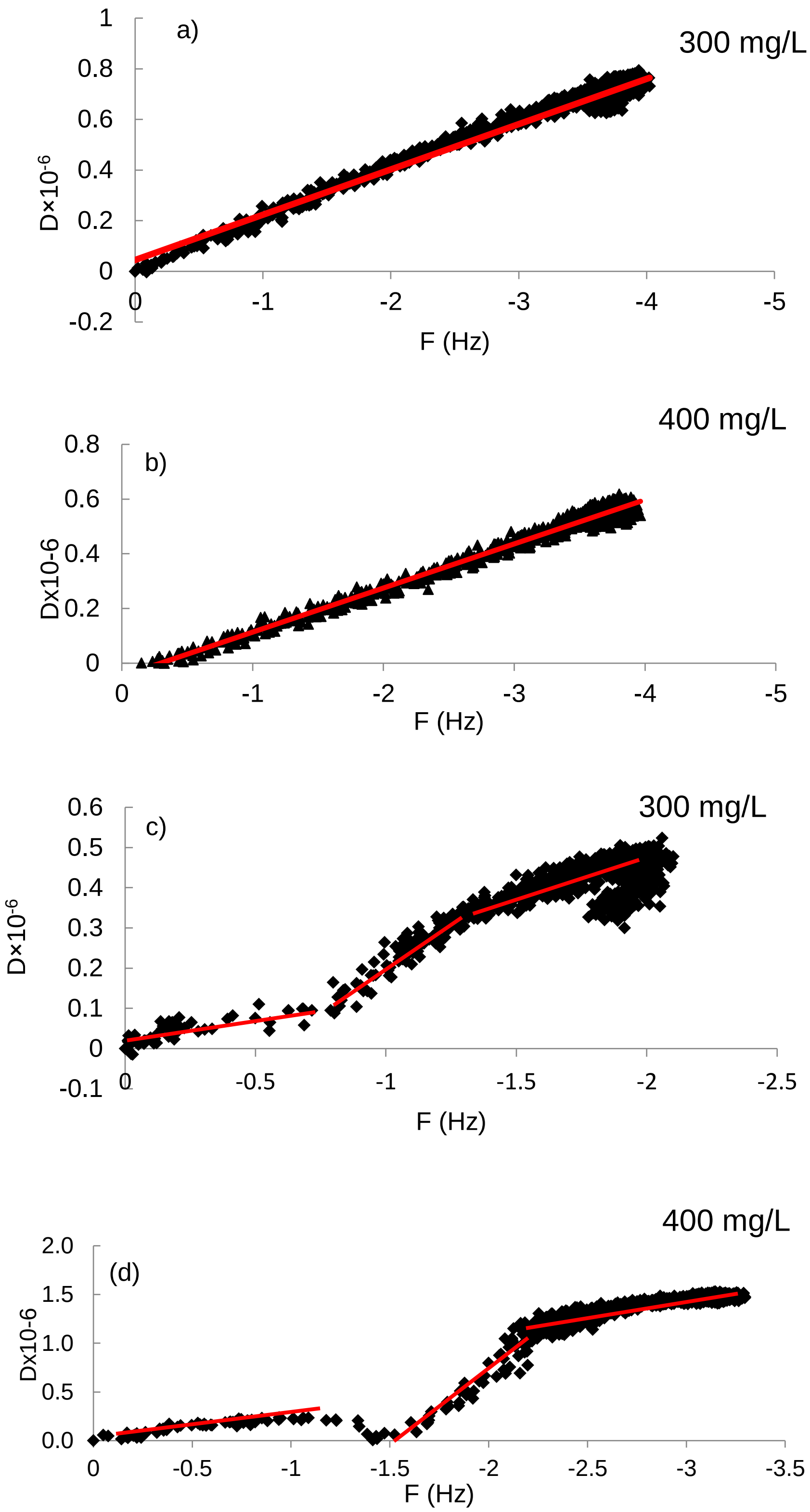

Figure S7. Dissipation change of the sensors as a function of frequency change for **a)** 300 mg/L SBL **b)** 400 mg/L SBL **c)** 300 mg/L SML **d)** 400 mg/L SML.

Table S4. Normalized dissipations together with correlation coefficients for lignin samples

	SBL	SML		
Concentration (mg/L)	K	K_1	K_2	K_3
300	0.18 (R^2 =0.97)	0.11 (R^2 =0.74)	0.42 (R^2 =0.82)	0.18 (R^2 =0.80)
400	0.16 (R^2 =0.98)	0.22 (R^2 =0.89)	1.58 (R^2 =0.92)	0.29 (R^2 =0.80)
500	0.20 (R^2 =0.92)	0.22 (R^2 =0.93)	0.59 (R^2 =0.90)	0.40 (R^2 =0.98)
